# Enabling Personalization for Digital Cognitive Stimulation to Support Communication With People With Dementia: Pilot Intervention Study as a Prelude to AI Development

**DOI:** 10.2196/51732

**Published:** 2024-01-16

**Authors:** Nick Hird, Tohmi Osaki, Samik Ghosh, Sucheendra K Palaniappan, Kiyoshi Maeda

**Affiliations:** 1 Aikomi Ltd Co Yokohama, Kanagawa Japan; 2 Faculty of Rehabilitation Kobe Gakuin University Kobe Japan; 3 Data Science and Engineering SBX Corporation Tokyo Japan

**Keywords:** dementia, digital technology, communication, engagement, cognitive stimulation, artificial intelligence, AI

## Abstract

**Background:**

Maintaining good communication and engagement between people with dementia and their caregivers is a major challenge in dementia care. Cognitive stimulation is a psychosocial intervention that supports communication and engagement, and several digital applications for cognitive stimulation have been developed. Personalization is an important factor for obtaining sustainable benefits, but the time and effort required to personalize and optimize applications often makes them difficult for routine use by nonspecialist caregivers and families. Although artificial intelligence (AI) has great potential to support automation of the personalization process, its use is largely unexplored because of the lack of suitable data from which to develop and train machine learning models.

**Objective:**

This pilot study aims to evaluate a digital application called Aikomi in Japanese care homes for its potential to (1) create and deliver personalized cognitive stimulation programs to promote communication and engagement between people with dementia and usual care staff and (2) capture meaningful personalized data suitable for the development of AI systems.

**Methods:**

A modular technology platform was developed and used to create personalized programs for 15 people with dementia living in 4 residential care facilities in Japan with the cooperation of a family member or care staff. A single intervention with the program was conducted with the person with dementia together with a care staff member, and for some participants, smell stimulation was provided using selected smell sticks in conjunction with the digital program. All sessions were recorded using a video camera, and the combined personalized data obtained by the platform were analyzed.

**Results:**

Most people with dementia (10/15, 67%) showed high levels of engagement (>40 on Engagement of a Person with Dementia Scale), and there were no incidences of negative reactions toward the programs. Care staff reported that some participants showed extended concentration and spontaneous communication while using Aikomi, which was not their usual behavior. Smell stimulation promoted engagement for some participants even when they were unable to identify the smell. No changes in well-being were observed following the intervention according to the Mental Function Impairment Scale. The level of response to each type of content in the stimulation program varied greatly according to the person with dementia, and personalized data captured by the Aikomi platform enabled understanding of correlations between stimulation content and responses for each participant.

**Conclusions:**

This study suggests that the Aikomi digital application is acceptable for use by persons with dementia and care staff and may have the potential to promote communication and engagement. The platform captures personalized data, which can provide suitable input for machine learning. Further investigation of Aikomi will be conducted to develop AI systems and create personalized digital cognitive stimulation applications that can be easily used by nonspecialist caregivers.

## Introduction

### Background

The lack of effective drugs for dementia [1] means that, for the foreseeable future, high-quality care remains the best option to maintain quality of life (QOL) for persons with dementia. However, cognitive decline and behavior changes associated with dementia increase the complexity and difficulty of caregiving, often making it challenging for families and care staff [2]. In particular, responsive behaviors, known medically as behavioral and psychological symptoms of dementia (BPSD), are a range of neuropsychiatric disturbances that affect most persons with dementia and can greatly disrupt caregiving, causing both poor QOL for the person with dementia and physical and mental stress for their caregivers [3]. Communication and engagement between people with dementia and their caregivers lie at the heart of good-quality caregiving [4,5], which is usually provided in dyadic or triadic structures [6] formed by the person with dementia and professional care staff and family caregivers. Communication plays a key role in the successful functioning and quality of care relationships in these care structures as well as greatly influencing the well-being and QOL of everyone involved [7]. Unfortunately, the progression of dementia can significantly impair the communication process for both persons with dementia and their caregivers and lead to inadequate or 1-sided interactions. Overcoming communication issues requires skill, patience, and sensitivity on the part of caregivers, which further adds to the difficulties and stress of caregiving [8]. In addition, the lack of time and resources available for caregivers can deprioritize communication as an activity in itself and result in it being conducted while performing other care activities, which may not be sufficiently personal or meaningful to maintain the psychological well-being of the person with dementia [9].

Communication and engagement are also integral to person-centered care [10], which has been shown to support QOL and help manage responsive behaviors and is widely accepted as best practice in dementia care. Communication difficulties between persons with dementia and their caregivers can prevent adequate expression and understanding of needs, desires, and intentions for both [11], presenting a major barrier to implementing person-centered care [12] including in Japan [13]. Communication and engagement are also important for implementing most psychosocial interventions [14], which are widely used to support caregiving activities to maintain well-being and QOL and manage BPSD. Many psychosocial interventions are based on different types of cognitive stimulation activities, such as cognitive training [15], reality orientation [16], reminiscence therapy [17], multisensory stimulation [18], and music therapy [19]. Several psychosocial interventions have shown promising clinical evidence, in some cases comparable with drug therapies, especially when implemented at the individual level. One of the most well-validated psychosocial interventions is cognitive stimulation therapy (CST) [20], which is based on person-centered care and consists of systematic protocols that combine reminiscence therapy and reality orientation designed to promote enjoyable and meaningful activities for persons with dementia. Clinical studies in groups have demonstrated improved cognition and QOL for persons with dementia [21] and improved caregiver relationships when conducted at the individual level (individual CST; iCST) [22]. Culturally adapted CST protocols have been developed for >20 countries, including CST-J for Japan [23], although its adoption in Japanese care settings remains limited, especially at the individual level.

The lack of trained care staff and practical difficulties associated with the regular and consistent implementation of psychosocial interventions have generated considerable interest in the use of digital technologies [24,25], a trend that was accelerated by the COVID-19 pandemic [26]. Recently, several applications have become available to promote personalized communication and engagement between people with dementia and their caregivers [27], and personalization has been recognized as an important factor in obtaining sustainable benefits [28]. Digital storytelling is a promising approach based on the well-established life story book concept in reminiscence therapy, which uses a person’s own and other relevant content to create fully individualized interventions [29]. In digital storytelling, the physical materials commonly used to facilitate life story book interventions, such as photographs, books, and memorable objects, are replaced with digital media, such as images, videos, and audio. In addition to engagement, digital storytelling aims to help the person tell their own story and has also been used outside dementia in other areas of mental health. Feasibility studies with digital storytelling applications have shown improvements in memory, QOL, and depression [30] as well as additional benefits from the use of digital media. Similar results have been observed in both Western and Asian contexts [31], indicating the wide potential of this approach. However, one of the difficulties of digital storytelling is that preparation requires digital skills, time, and effort [32], which presents a significant adoption barrier for many caregivers and families. The need to create personalized content is avoided through a digital iCST application [33] that uses a pool of precreated generic stimulation activities, including quizzes and games designed to promote engagement, that can be used according to the interests and preferences of the users. A feasibility study showed promising results [34]; however, the lack of personalized content and content diversity was identified as an issue for maintaining engagement and long-term use. Given the importance of personalization, reducing the time and effort required for tailoring applications is a key concern to facilitate broader adoption by nonexpert care staff and families. Data generated by digital technologies can be used to develop artificial intelligence (AI) systems to support caregiving for dementia [35], which can aid the personalization process. However, the development of personalization applications is still at an early stage, and the lack of high-quality personalized data sets related to communication and engagement for people with dementia hinders progress in this area. To address this, a prototype application called Aikomi was developed to support communication with people with dementia while also capturing high-context personalized data, including behaviors, that would provide a suitable source for developing machine learning models to automate the personalization process. Such AI systems could reduce the personalization burden of digital cognitive stimulation applications and enable their use by nonspecialist caregivers to support communication and engagement with people with dementia in real-world settings.

### Aims and Objectives

The aim of this pilot study was to evaluate the Aikomi application in Japanese residential care homes for its potential to (1) create and deliver personalized cognitive stimulation programs and promote communication and engagement between people with dementia and their usual care staff and (2) capture meaningful personalized data suitable for the development of AI systems.

## Methods

### Technology Development

The Aikomi application was designed by a multidisciplinary team with expertise in occupational therapy (TO); clinical dementia care and psychiatry (KM); digital health (NH); engineering, data science, and machine learning (SG and SKP); the design process also received input from frontline care staff in Japan. The design goal was to create an application that could be routinely used by care staff and family members to promote communication with people with dementia living in both residential care and community settings and would not require expert knowledge, training, or long preparation times. The technology goals were to (1) construct the technology platform using a modular architecture with stand-alone applications for each key function to allow for “plug-and-play” integration of software and hardware applications and (2) incorporate an open connectivity platform to allow for convenient data flow between application modules and facilitate the development of machine learning applications. The connectivity platform used was Garuda (Garuda Alliance) [36], which is a community-driven open connectivity platform previously developed by one of the authors (SG). The modular design provides flexibility to develop fit-for-purpose applications as well as “future-proof” the platform to incorporate other technologies and data sources to enhance optimization and scaling of the personalization process.

**Figure 1:**
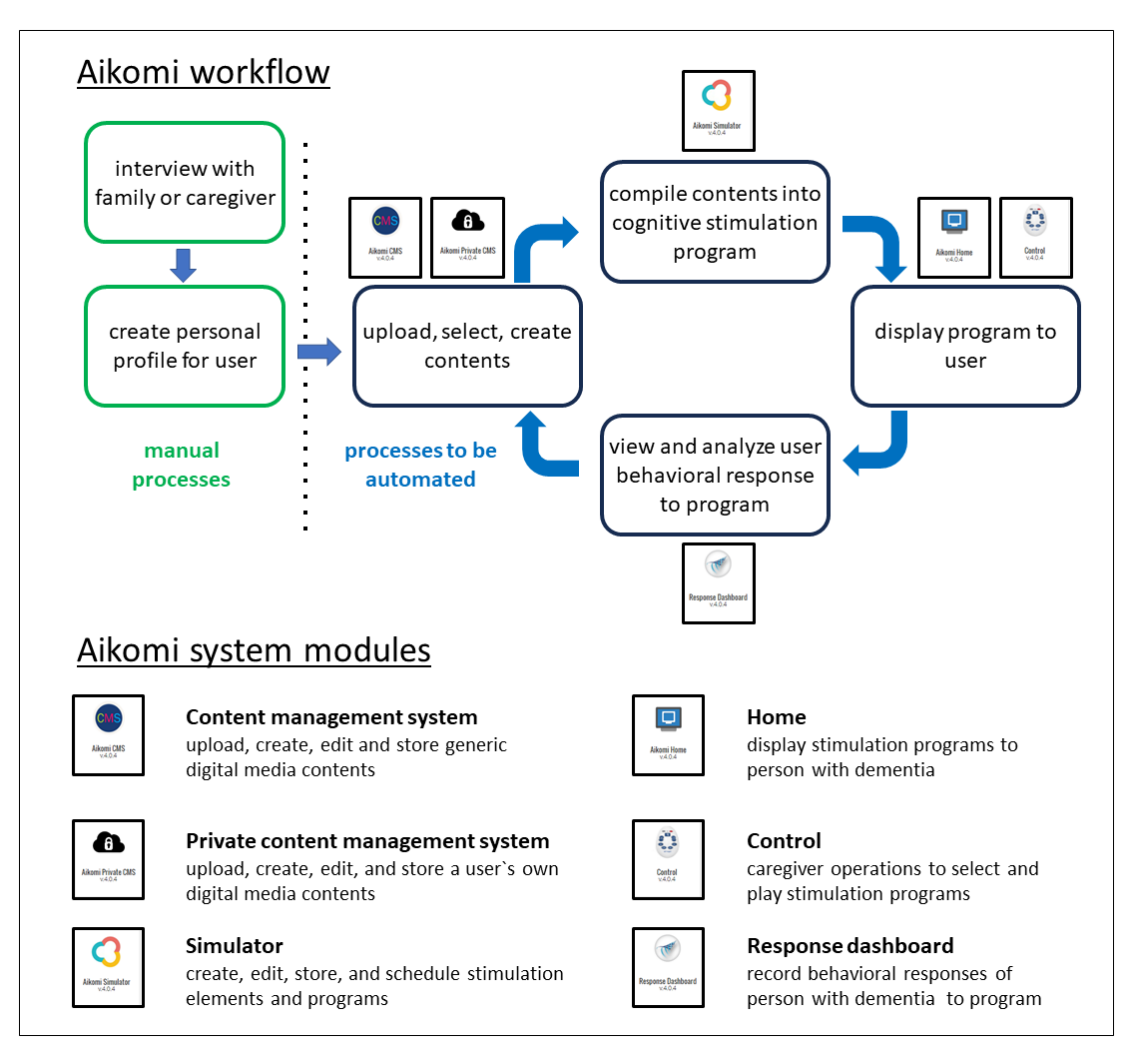
Aikomi workflow and system modules.

A 6-step workflow for using the application was designed through informal discussions with professional care staff and families (Figure 1). The first step is to conduct an interview with families or care staff to obtain information about the person with dementia, including their life history, hobbies, interests, preferences, and abilities, which are used to create a standardized personal profile. On the basis of this personal profile, relevant digital media content is selected or created, and if available, the person’s private content obtained from the family is digitized (as necessary) and uploaded to the system. Next, relevant content is compiled into audiovisual stimulation programs, which are displayed as the intervention. Finally, the behavioral responses of the person with dementia during the intervention are recorded and analyzed to adapt and optimize the content and stimulation programs for the next intervention. To minimize the burden of the personalization process on families and care staff, their participation is limited to the initial interview and the intervention itself. The remaining personalization processes were conducted manually by the research team for this pilot study and will be automated in the future.

**Figure 2:**
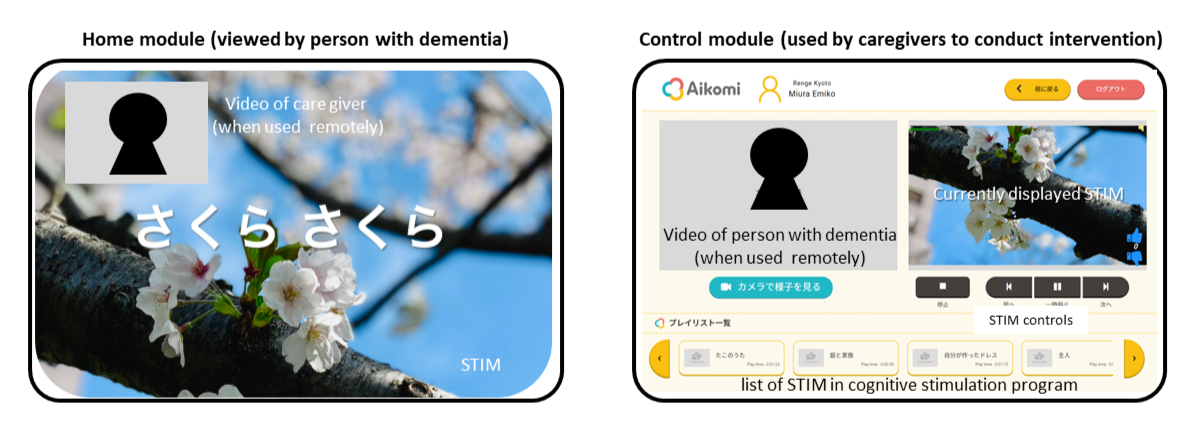
Home and control module display.

The software for all the components (modules) was developed as an independent web application whose integration was facilitated by the Garuda connectivity platform. The content management system (CMS) is a repository for generic digital media content, such as images, videos, and audio files. It allows users to upload, create, edit, and store content that can be used by all persons. A separate private CMS module performs the same function as the CMS for the private content of the person, but its use is restricted to this person only. The simulator module is a function to create stimulation elements (called STIMs) from the content in the CMS and private CMS. A STIM consists of a short audiovisual sequence created from combinations of image, audio, and video data and is the building block used to create stimulation programs. The user has complete flexibility to create and select STIMs and compile them in any sequence order to generate the stimulation program. The personal CMS and simulator modules operate at the level of each individual and are not accessible to other users and caregivers. The simulator module also includes a function to create and edit a standardized personal profile of the person. The home and control modules are both used to conduct the intervention. The home module displays the stimulation programs viewed by the person with dementia, and the control module is used by the caregiver to select and control the display of the stimulation program on the home module during the intervention. The control module has functions to pause the program to talk or go back to a previous STIM or forward to a new STIM depending on the response of the person with dementia. A web meeting function was integrated into the home and control modules to allow for the remote use of the Aikomi application, but it was not used in this pilot study, in which only in-person sessions were conducted. The design of the functions and user interfaces for the home and control modules was conducted in collaboration with care staff primarily for use with tablets, but they can also be used on a PC. An example of the home and control modules during use is shown in Figure 2.

The response dashboard module enables the storage, review, and data analysis of video recordings of the person with dementia during the intervention. It was intended for video recordings to be made using the camera on the home tablet; however, this function was not fully operational at the time of the pilot study. Hence, a remote camera was used to record the interventions, and the data were subsequently uploaded to the response dashboard. Each module was a separate web application integrated with the Garuda connectivity platform. The tablets used in the pilot study were installed with SIM cards to avoid the need to use the local network at the residential care home, which can sometimes be unreliable.

### Pilot Study

#### Study Design and Participants

The study was conducted using Aikomi as a single intervention lasting approximately 30 minutes with 15 people with dementia living in 4 urban residential care homes in Japan and their usual care staff. The participants were nominated by the care managers at each facility based on the following selection criteria: (1) a diagnosis of dementia, (2) displaying negative BPSD such as anxiety or apathy, (3) no hearing or visual difficulties that would prevent using a tablet, and (4) agreement from their family to participate in the study. Participants with a diagnosis of frontotemporal dementia or who had other mental conditions were excluded from this study. All the care staff members who participated in this study were qualified professionals at the residential facilities.

#### Procedure

**Figure 3:**
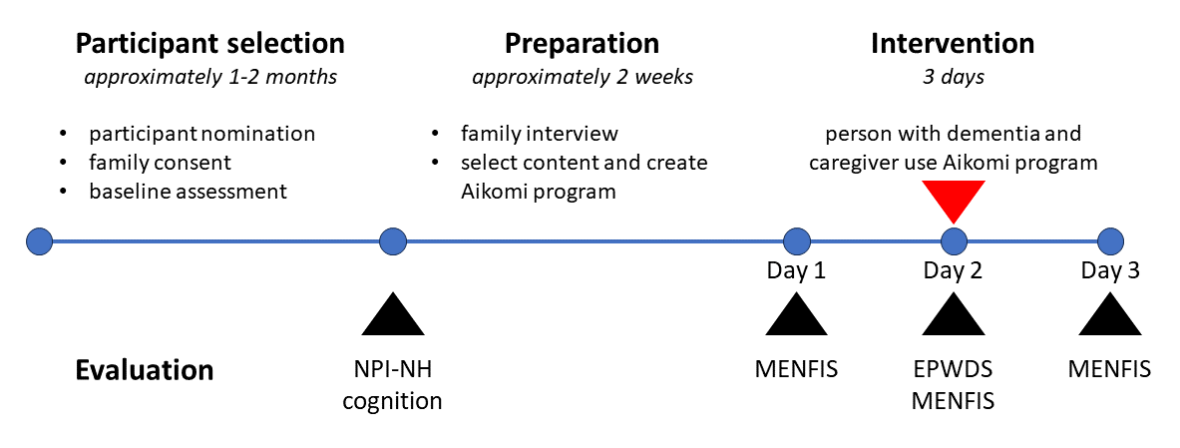
Pilot study plan. EPWDS: Engagement of a Person with Dementia Scale; MENFIS: Mental Function Impairment Scale; NPI-NH: Neuropsychiatric Inventory–Nursing Home version.

The protocol and timeline for the pilot study are shown in Figure 3.

#### Participant Selection

Care managers at participating care homes explained the details of Aikomi and the pilot study to the families of candidate persons with dementia using materials provided by the research team. After the explanation, written consent was obtained from the families who agreed to participate in the study.

#### Program Preparation

The research team conducted in-person or telephone interviews with family members to obtain information about the person’s life story, interests, and preferences as well as other topics that the family thought could be meaningful. In addition, the families were asked to provide any suitable family photos if they had them. In one case, a family member was not available for interview, and it was instead conducted with the care manager at the facility. The obtained information was used to create a profile for each person, and relevant digital content was selected and used to create STIMs that corresponded to items in the profile, such as hometown, childhood, family, work, life and cultural events, hobbies, sports, travel, pets, and music. The duration of each STIM ranged from 30 seconds to 3 minutes, and each stimulation program was created by compiling 10 to 20 STIMs in a sequential order expected to be easy for the person with dementia to follow. For this pilot study, all non–family-derived content was obtained by the research team from publicly available sources, and the programs were prepared by the research team within approximately 2 weeks following the family interview.

#### Intervention

The intervention was conducted as a 1:1 session with the person with dementia and the care staff member seated next to each other at a table in a quiet area of the care home. The home tablet was placed at a comfortable viewing distance for the person with dementia and the care staff member. The control tablet was operated by a research team member seated on the opposite side of the table. A camera was placed at an appropriate position to record the behavioral responses of the person with dementia and care staff member during the intervention. As the application was to be operated by the research team, the care staff were only given a brief overview of the device, and a rehearsal was conducted before the session to familiarize them with the intervention conditions. In addition, to allow the person with dementia to lead responses to the stimulation program as much as possible, the care staff were requested to adopt a passive role during the intervention and respond appropriately according to the person’s behavior and mood, although prompting according to their own judgment was permitted. The stimulation program was started as soon as the participant was seated and appeared comfortable, and apart from initial greetings, the research team member avoided direct interaction with the person with dementia during the session unless actively addressed by them. The intervention began with a few general reminiscence STIMs to act as a “warm up” and accustom the person with dementia to the device, after which STIMs related to personalized topics were played. The selection of STIMs was adjusted according to the mood and responses of the participant, and if a participant showed good engagement with a particular theme, the STIM was repeated or a related STIM was shown. In cases in which the participant showed little engagement, the STIM was stopped and a STIM of a different topic was shown. The target time for each intervention was 30 minutes but was shortened if the participant appeared tired or uninterested, and the STIM was changed immediately if the participant appeared uncomfortable. The intervention was concluded by showing STIMs such as nature or landscapes as a “cool down” period, and if the participant was agreeable, smell stimulation was conducted using 5 synthetic smells that were embedded on paper strips (such as those used for sampling perfumes) supplied by a commercial smell product company [37]. The decision to use the smell sticks was made after asking each person with dementia and the care staff member after completion of the main stimulation program. The 5 smells used for the evaluation were chocolate, miso (fermented soy paste cooking ingredient), grass, earth, and soap. A smell stick was provided to both the participant and the caregiver without revealing the identity of the smell, and at the same time, a STIM related to the smell was displayed on the home module, for example, showing a bar of a famous brand of chocolate for the chocolate smell. Following the first smell, the remaining smell sticks were similarly used individually according to the participants’ interest in continuing.

### Evaluation Scales

#### Baseline

BPSD assessment of the participants was conducted during the month before the intervention by the appropriate care staff using the Neuropsychiatric Inventory–Nursing Home version (NPI-NH) [38]. Cognitive ability was recorded using the most recent cognitive assessment conducted at the care home using one of the following cognitive scales: Mini-Mental State Examination (MMSE) [39], revised Hasegawa Dementia Scale (HDS-R) [40], or Nishimura Mental State Scale for the Elderly (NM) [41]. In total, 3 different cognitive scales were used because of the different types of cognitive tests routinely used at each participating care home.

#### Intervention

Engagement during the intervention was measured using a Japanese translation of the Engagement of a Person with Dementia Scale (EPWDS) [42] prepared by the research team. The EPWDS is a 10-item assessment measuring positive and negative engagement in 5 domains—affect, visual, verbal, behavioral, and social—and was administered soon after the intervention by the attending care staff member, who was also asked to provide their comments on the intervention. Psychological well-being was measured using the 6 motivational and emotional dysfunction items of the Mental Function Impairment Scale (MENFIS) [43]. The MENFIS assessment was conducted at the care home by a care staff member on 3 consecutive days spanning the intervention: the day before the intervention (day 1), on the day of the intervention a few hours after it was conducted (day 2), and the day after the intervention (day 3). The care staff member was also asked to provide their written comments for each assessment. All 3 MENFIS assessments were conducted by the same care staff member, who was not always the same care staff member who was present at the intervention. As the study was intended as a preliminary pilot study, the EPWDS and MENFIS scores were only used as a guide for the qualitative assessment of the intervention.

### Ethical Considerations

Ethics approval was obtained from the Kobe Gakuin University Human Research Ethics Committee (sourin 18-14). Written approval to conduct the study was obtained from the directors of each of the participating care facilities as well as the families of all the persons with dementia participating in the study.

## Results

### Overview

The characteristics of the 15 participants enrolled in the study are shown in Table 1. A total of 5 (33%) people did not meet all the selection criteria: 2 (13%) were “suspected” to have Alzheimer disease but had not received a formal diagnosis of dementia, and 3 (20%) did not show BPSD as defined by the NPI-NH. However, it was decided to include all 5 in the pilot study as the primary purpose was to evaluate Aikomi for its acceptability and communication effects on persons with dementia, and there was agreement to proceed from the care staff and families. The people who did show BPSD were reported to have a wide range of symptoms.

**Table 1:** Baseline characteristics of persons with dementia in the pilot study.

Person	Care home^a^	Sex	Age (y)	Dementia type^b^	Cognitive scores				BPSD^c^		Care level^d^
					HDS-R^e^	MMSE^f^	NM^g^	NPI-NH^h^			
1	A	Male	93	AD^i^	8	17	ND	0		IIIa	
2	A	Female	89	AD	7	11	ND	12		IIb	
3	A	Female	86	AD	2	ND	ND	20		IV	
4	A	Female	86	AD	ND	21	ND	0		IIIa	
5	B	Male	79	AD	ND	24	ND	36		IIb	
6	B	Male	76	AD	ND	8	ND	26		IIIb	
7	B	Female	87	AD	ND	17	ND	7		IIb	
8	B	Female	84	AD	ND	16	ND	10		IIb	
9	B	Male	79	AD	ND	20	ND	16		IIb	
10	B	Female	83	AD	ND	9	ND	17		IIb	
11	B	Female	86	AD	ND	15	ND	4		IIb	
12	C	Female	90	AD	6	ND	ND	36		IIIb	
13	C	Female	86	AD	0	ND	ND	4		Ia	
14	D	Female	77	ND^j^	ND	ND	6	6		IIb	
15	D	Female	90	ND	ND	ND	25	27		IIIa	

^a^A: group home; B: residential nursing home; C and D: older adult rehabilitation facility.

^b^Reported dementia diagnosis.

^c^BPSD: behavioral and psychological symptoms of dementia.

^d^Japanese long-term care insurance rating scale [44].

^e^HDS-R: revised Hasegawa Dementia Scale [40].

^f^MMSE: Mini-Mental State Examination [39].

^g^NM: Nishimura Mental State Scale for the Elderly [41].

^h^NPI-NH: Neuropsychiatric Inventory–Nursing Home version [38].

^i^AD: Alzheimer disease.

^j^ND: not determined.

### Engagement

The responses of the persons with dementia during the intervention are shown in Table 2 and Multimedia Appendix 1. The duration of the intervention ranged from 15 to 38 minutes, most persons (13/15, 87%) showed strong positive responses to at least 1 of the STIMs, and none showed discomfort toward Aikomi or requested the session to be stopped. In total, 67% (10/15) of the participants had an EPWDS score of >40 (out of a maximum of 50), indicating both a high incidence of positive engagement and a low incidence of negative engagement. The remaining 33% (5/15) of the participants showed an EPWDS score of >30, which was due to low positive engagement scores (lack of engagement with the stimulation program) rather than the high incidence of negative responses such as anger, anxiety, or discomfort, which were not observed for any participant. Interestingly, person 14, who showed few positive responses during the intervention itself, spoke to thank the research team after the session was completed, which the care staff member said was highly unusual behavior for them.

The types of STIM topics and the responses they generated were analyzed from the video recordings and are shown in Table 3. The most common STIM topic to generate good engagement was family photos, which prompted self-initiated talk and the identification of persons they recognized. However, some participants (6/15, 40%) struggled to recognize their family members and even themselves. One person (person 3) showed no response, and person 4 responded most strongly to photographs of herself in early adult life rather than of her family. Music was also a popular STIM topic, prompting several participants to initiate singing and clapping to both traditional Japanese children’s songs (*doyo*) and Japanese popular music. The STIM of popular singers known to be liked by the persons with dementia often generated verbal dialogue with the care staff (persons 4, 6, 8, and 13). Japanese traditional arts, such as the tea ceremony, dance, and calligraphy, generated good responses from most women who had performed them (persons 2, 4, 7, 8, 11, and 13), and sports themes (baseball, sumo, and boxing) were popular for all the men (persons 1, 5, 6, and 9). Work-related themes induced mixed responses; however, STIMs related to actions performed during their work (eg, using a Japanese typewriter, counting money, or preparing fish) generated responses from 33% (5/15) of the participants (persons 2, 6, 7, 8, and 15). Wartime navy service STIMs generated strong positive engagement from person 1 and prompted a detailed recollection of his experiences; this is described in more detail in case study 1.

**Table 2:** Engagement during the session with the Aikomi device.

Person	EPWDS score^a^	Caregiver written comment provided after the session (translation from Japanese)^b^
1	46	He showed very spontaneous reaction to family and the warship.
2	33	She concentrated for volleyball and knitting but could not remember family faces or names. She has severe memory loss.
3	37	She quickly responded to music, but the response is similar to what the care staff can obtain using tablets.
4	41	She was able to concentrate due to the music, and was very focused for one singer. Usually, her concentration doesn’t last for 5 minutes, it was very unusual for her to maintain concentration for 30 minutes.
5	48	He turned to look at the tablet as soon as the images appeared and focused on talking about them, including in great detail about the movies. This is behavior not usually observed by the care staff.
6	34	Despite being a shy person, he was able to sing in front of everyone. He usually can’t recognize things, but was able to for some pictures. He was anxious because he couldn’t understand many things.
7	42	She did hand gestures to songs and Japanese dance and hula dance. She did not say much because she is a naturally reserved person, but she showed concentration and seemed excited.
8	39	She looked at the tablet and talked continuously but not related to the themes shown. She showed good responses to music and smell.
9	46	He talked about his mother and explained to us in detail about his old hobbies. He was anxious because he was able to detect the smells.
10	45	She showed good expression and mood. Usually she doesn’t continue laughing, and I think it was due to the continuous stimulation.
11	45	She clapped her hands and sang to the music, which is the same response as she regularly shows with karaoke.
12	32	She had pneumonia before the test, which reduced her will, and she only responded weakly to themes.
13	40	She is usually not a person who can show good concentration but was calm and concentrated during the test and could recall her memories.
14	40	She showed most interest in the old photos. At the end of the test, she smiled and said “thank you”.
15	47	She sang along to the music and looked nostalgic when watching the old photos.

^a^EPWDS: Engagement of a Person with Dementia Scale. Range of scores is 10-50, higher scores indicate higher level of positive engagement, lower scores indicate higher levels of disengagement or negative engagement [42].

^b^Translation by the research team.

**Table 3:** Engagement by stimulation element (STIM) topic.

Person	Self-initiated talk^a^	Prompted talk^b^	Notes^c^
1	Family, navy service	Hometown, baseball, photography	Viewed war service STIM several times, adding new anecdotes each time. Responded strongly to family photos.
2	Volleyball, Japanese typewriter, kimono	Family, music, cooking, childhood	Husband of participant 1 but did not respond to family photos. Was good at volleyball. Used typewriter at work.
3	None	Music	Only responded to music (sang). Did not react to personal topics.
4	Japanese singer, photos (self)	Family, music, Japanese dance	More interested in childhood and own early adult life photos than in family photos. Explained about Japanese dance. Sang to music.
5	Archery, 100-km walk, food, pigeon racing, gardening, childhood	Family, school, life, pet, hot spring bath, hometown	Talked in detail about participating in 100-km walk. Said that gardening STIM showed incorrect way to grow orchids. Talked about difficult times during childhood.
6	Boat race, Japanese singer, sumo, food	Baseball, cooking fish, family, music, cultural event	Nervous at first but calmed down when the program started. Talked about working at a fish restaurant. Did not recognize many family members. Sang to music.
7	Family, Hawaii, and Japanese arts	Bank, counting money	Talked about cousin who lived in Hawaii. Remembered how to count money. Looked closely at calligraphy and flower arranging.
8	Yakitori (grilled chicken), dog, Japanese poetry	Japanese singer, family, music, hometown	Talked continuously but not related to STIM. Stopped talking to look at yakitori and Japanese poetry. Sang to music.
9	Family, movies, boxing, baseball	Detective novel, dog	Talked about family life and children and in great detail about films, naming actors and directors. Identified boxers and baseball players and talked in detail.
10	Watercolor painting, hill walking, athletics	Family, knitting, television drama, food	Said that she wanted to try watercolor painting and knitting. Named some mountains she had climbed. Said she was fast at running at school.
11	Family, hometown, music, tea ceremony	Schools, school sports, music, cooking	Could identify more family members on repeated viewing. Repeated name of hometown several times. Watched tea ceremony closely for several minutes. Sang to music.
12	None	None	Did not look at the screen for most of the session. Showed no reaction to any STIM.
13	Hometown, Japanese singer, *koto* (Japanese musical instrument), childhood	Kobe, childhood songs	Talked about growing up in Tokyo and sad life of Japanese singer. Watched *koto* playing closely for several minutes.
14	None	None	Looked continuously at the tablet but did not show any reaction to any STIM
15	Family, travel, childhood, hotel work	Paper making, hot spring bath, music, son’s work	Talked about family and climbing Mount Fuji with her son. Recalled working at a hotel and her son’s company.

^a^Talk initiated by the person with dementia without prompting.

^b^Person responded to prompting by the care staff member.

^c^On the basis of a video review by the research team.

### Smell

A total of 60% (9/15) of the participants tried the smell stick stimulation in conjunction with paired audiovisual stimulation, and 13% (2/15) of the participants tried all 5 smell sticks. The responses are shown in Table 4. In most cases, the smell sticks led to pleasurable responses from both the participant and the care staff member, with more laughter than was observed during the audiovisual programs. Almost all the people with dementia (8/9, 89%) had difficulty explicitly identifying the smell; however, majority (6/9, 67%) were able to notice that the smells were different, and recognition of the smell identity did not appear to affect the engagement with the caregivers. Although it was not the intention, some participants perceived the smell program as a test, which may have caused some confusion (person 15) and prompted one person to say that she had lost her sense of smell (person 5). This shows that care must be exercised when using smell stimulation to reduce the risk of causing anxiety to users who may no longer have a sense of smell or be stressed by the inability to correctly identify the smells.

**Table 4:** Response of participants to smell sticks combined with audiovisual stimulation.

Person	Smell stick used						Response^a^
	First	Second	Third	Fourth	Fifth		
1	Chocolate	Soap	—^b^	—	—	Smelled the stick and, when prompted, looked at the picture. Did not identify either smell verbally but said the name of the chocolate brand and soap brand shown in the pictures in each case. Appeared to enjoy the experience.	
3	Chocolate	Miso	—	—	—	Smelled the stick and said that she could not smell anything for chocolate but could smell something for miso.	
5	Chocolate	Soap	—	—	—	Said that she could not smell either smell and, unprompted, said that she had lost her sense of smell.	
8	Chocolate	Soap	Grass	Miso	—	Said that she liked the smell and talked about chocolate. Recognized the second smell as soap and said she liked it. Noticed that the third smell was different but could not identify it. Said that the fourth smell was a food smell but she did not know what it was. Talked and laughed during the smell session.	
9	Chocolate	Soap	—	—	—	Noticed that the first stick had a smell and then said that it smelled delicious and talked about the picture. Did not notice the second smell and said that his sense of smell was poor. Talked and laughed during the smell session.	
10	Chocolate	Soap	Grass	Earth (soil)	Miso	Very actively smelled the first stick and laughed but did not identify the smell. Noticed that the second smell was different. For the third, fourth, and fifth smells, the participant actively smelled the sticks but said that they smelled the same. Talked and laughed during the session.	
11	Chocolate	Soap	Earth (soil)	Miso	Grass	Actively smelled the first stick and asked what it was. Said “not bad” to the second and third stick and noticed that all smells were different but could not recognize them when looking at picture prompts. Talked and laughed during the session.	
13	Chocolate	—	—	—	—	Smelled the stick but said that it had no smell.	
15	Chocolate	Soap	—	—	—	Smelled the first stick but said that she could not smell anything. Appeared reluctant to try the second smell.	

^a^On the basis of a video review by the research team.

^b^Not used.

### Well-Being and Behavior Changes

The results of the MENFIS well-being assessment of the persons with dementia conducted by the care staff over the consecutive 3-day intervention period are shown in Table 5. In total, 13% (2/15) of the participants showed improvement (reduction in MENFIS score) on the third day compared with the first, and 7% (1/15) of the participants (person 13) showed a reduction only on the second day (after the intervention). All other participants showed similar or identical MENFIS scores for all 3 days except for person 12, who showed worsening over the 3 days, which was attributed to her catching a cold during the period of the intervention. The data for persons 14 and 15 were disregarded as the care staff member mistakenly conducted the second-day assessment before the Aikomi session instead of after. In many cases, the participants had no recollection of the intervention the following day. Little inference can be drawn from the MENFIS data except that there appeared to be no adverse effects on the well-being of the people with dementia after using the Aikomi application.

**Table 5:** Well-being and behavior changes over the course of the intervention.

Person	MENFIS score^a^				Care staff written comments (translation from Japanese)^b^
	Day 1	Day 2	Day 3		
1	18	12	9	BPSD^c^ disappeared just before the test.	
2	26	23	21	Severe memory impairment but able to remember some things with repetition, and their self-confidence improved.	
3	24	11	14	Although apraxia and agnosia increased, her emotions were maintained.	
4	30	28	26	Her expression was inhibited by her DLB^d^ and psychotropic medication.	
5	13	13	13	No comments given.	
6	11	11	11	No comments given.	
7	10	10	10	No comments given.	
8	9	9	9	No comments given.	
9	14	14	14	No comments given.	
10	13	13	13	No comments given.	
11	3	3	3	After the test, she showed a different expression from usual and recalled that she had done something different.	
12	22	26	28	Day 1: usually does not express emotions except sometimes when singing; day 2: no change; day 3: as usual.	
13	27	12	27	Day 2: she said herself that she felt good, and other people said that she was not as angry as usual.	
14	24	27^e^	25	She laughed when she talked about the old photos.	
15	10	16^e^	13	She did not remember the test.	

^a^MENFIS: Mental Function Impairment Scale (6 noncognitive items). Day 1: evaluation the day before the session; day 2: evaluation on the day of the session after it was conducted; day 3: evaluation the day after the session.

^b^Translation by the research team.

^c^BPSD: behavioral and psychological symptoms of dementia.

^d^DLB: dementia with Lewy bodies.

^e^Recorded before the session was conducted.

### Selected Case Studies

#### Person 1

Although not formally experiencing BPSD according to the NPI-NH, the care staff member said that person 1 usually displayed apathetic behavior, and the staff member was very surprised by the amount of self-initiated dialogue in response to the family and wartime navy service STIM, which was shown several times during the test. The STIM included a photograph of the ship he served on as well as a Japanese navy song. The person became conversational and could recall the names of former navy colleagues as well as events in detail, providing new information and anecdotes with every repetition of the STIM. The care staff used this information to continue to manually provide stimulation using photographs on the following day, which is likely to have contributed to the continuing improvement trend observed in the MENFIS score from the second to the third day. In contrast, person 2, who was his spouse and lived at the same facility but showed more advanced dementia symptoms, did not respond strongly to the same family STIM used for person 1. However, at the request of the care staff member, an additional session with the Aikomi application was conducted with both persons 1 and 2 watching the family STIM together. In this case, person 1 provided prompts to person 2, who showed a much stronger response than when she viewed the STIM with the care staff member.

#### Person 10

The caregiver reported that the person had shown agitated behavior in the period before the intervention but was able to come into the room and quickly settled when the session started. She responded to several of the STIMs and actively talked about several of the themes presented. As she was an excellent athlete during her school days, the program included a STIM video of a modern high school girls’ 100-meter race, in which she showed great interest, including making a humorous comment about the lack of clothes worn by current athletes. In addition, she showed quiet concentration on the STIM related to her hobbies of watercolor painting and hill walking and told the care staff member that she wanted to do both again and recalled the names of several mountains that she had climbed. During the smell intervention, she laughed and engaged with the care staff member and research team and appeared to enjoy the experience even though she did not recognize any of the smells. The care staff member said that her behavior during the intervention was not usual and that she was not agitated when she returned to her living area. However, this was not reflected in the MENFIS scores, which were assessed by a different care staff member and showed no changes over the 3-day period.

#### Person 13

During the intervention, she showed good responses to STIMs about her hometown (Tokyo), her hobby of playing the *koto* (musical instrument), and a popular Japanese singer. She momentarily cried on 2 occasions when viewing her hometown and the Japanese singer, but her sadness did not persist and was followed by self-initiated talk to the care staff member about her wartime childhood and the Japanese singer’s unhappy life story. The care staff member reported that she usually could not show good concentration, and they were surprised that she was calm and could concentrate during the intervention and recall her memories. In the evening after the test, the care staff member reported that she said that she felt good and that other residents noticed that she was not as agitated as usual; however, on the day after the test, her agitated behavior returned to normal, and this pattern was reflected in the MENFIS scores.

## Discussion

### Principal Findings

Maintaining communication and engagement for persons with dementia is integral to caregiving, implementing person-centered care, and managing BPSD. It is also vital for facilitating the positive aspects of care that foster good QOL and sustainable caregiving relationships [45]. Over the last decade, several digital applications have become available to support communication and engagement for people with dementia [27], and to the authors’ knowledge, this pilot study is the first investigation of a digital application for personalized multi-sense stimulation conducted in Japan. All participants in this study were able to accept viewing the programs on the tablet, and these preliminary findings are broadly consistent with those of previous studies that demonstrate the importance of tailoring cognitive stimulation content to the individual profile and abilities of each person to obtain good engagement [28]. As reported for other applications, themes based on personal photographs or music often generated positive responses from participants, although for many people in the study, these themes did not generate the strongest responses during the intervention (Table 4). Instead, a wider range of themes specifically associated with the person’s lived experiences, such as wartime service (person 1), and interests such as gardening (person 5) and Western movies (person 9) were often the ones that resulted in prolonged and in-depth participant-initiated communication with care staff. This is in agreement with digital storytelling studies that have demonstrated the need to use a diverse range of relevant topics to adequately personalize interventions to obtain good engagement [29,30]. In addition, the benefits of using applications that combine both generic activity-promoting content and personally sourced content have been demonstrated with the CIRCA and CIRCUS application [46]. The role of the care staff was important to facilitate engagement in this study, especially to allow persons with dementia sufficient time to respond to the stimulation. It has been reported that caregivers sometimes do not allow sufficient time for the person with dementia to respond, which can lead to carer-directed engagement and suppression of the person with dementia’s own ability to initiate and maintain conversation [47]. In several cases, participants did not respond immediately to the stimulation content and required some time to view and “acclimatize” to the content before responding. This suggests that adapting the duration and complexity of the STIM according to the responses of each person may enhance their ability to respond and avoid premature transition to the next STIM, which will be explored in the future. The example of a husband and wife (persons 1 and 2) suggests the potential of people with dementia using the Aikomi application together without the active presence of care staff when the content has a high personal meaning for both. In total, 13% (2/15) of the participants, who had advanced dementia (persons 12 and 14), showed almost no engagement; however, good engagement was observed with another 13% (2/15) of the participants, who had low cognition (persons 6 and 10), suggesting that other factors in addition to cognition, such as content relevance, mood, and care staff behavior, may also be important for engagement.

One of the features of the Aikomi platform is that it allows for flexible navigation through a sequence of personalized stimulation themes that provide options for caregivers to select and adapt the content of the stimulation program according to the mood and responses of the user. The usefulness of this function was illustrated in person 1, who showed an unexpectedly strong reaction to the war-themed pictures triggering extended dialogue with the caregiver that was facilitated by several repetitions of the same STIM. In contrast, when person 13 started to make negative comments while viewing a STIM, it was possible to quickly change to a new STIM about her hobby, after which she started to make positive comments. Furthermore, the ability of the Aikomi application to easily modify and add new content enables the convenient creation of new personalized stimulation programs each time the Aikomi device is used. This is important for longer-term use to minimize or avoid the repeated use of the same content, which may lead to reduced interest from users and caregivers and has been found to be an issue for applications restricted to a fixed pool of content [33]. In addition, the flexible use of personally targeted activity content has been reported to promote curiosity and encourage self-directed learning in people with dementia [48] and suggests that personalizable digital cognitive stimulation applications such as Aikomi may be able to expand the range and depth of self-expression of people with dementia.

A secondary objective of this study was to explore the potential benefits of using paired smell and audiovisual stimulation to promote improved engagement compared with audiovisual stimulation alone. Currently, the use of smell stimulation in dementia care is limited and mainly focused on aromatherapy approaches to improve mood and reduce responsive behaviors using natural oils. However, other studies have shown that smell may play a role in triggering autobiographical and implicit memory [49], and it was thought that synthetic smells associated with daily life experiences could encourage not only the person with dementia but also the care staff member to share their own experiences, as well as being enjoyable. The engagement during paired smell and digital stimulation appeared to involve more smiling and laughter for both the participant and care staff member compared with responses to the audiovisual program alone. Interestingly, this was the case even when the person with dementia did not identify the smell and suggests that the combined use of digital audiovisual stimuli with smell may be more effective than smell alone [50].

From its inception, the Aikomi platform has aimed to develop AI capabilities to minimize the time and expertise required by nonspecialist care staff and families to use personalized digital applications. This is a critical issue for adoption and sustained use in dementia care, where caregivers are often older and not “digital natives” and have limited time or support to learn how to use and personalize applications [51]. Currently, most digital applications support personalization using two types of approaches: (1) supported collaboration with families to create bespoke interventions or (2) provision of predesigned content for on-demand selection during application use [28]. The bespoke approach often focuses on personal reminiscence and identity-reinforcing applications such as digital storytelling [29,30] and memory books [52,53], but preparation requires extensive family involvement over weeks or months, which may be challenging to sustain. Conversely, on-demand selection approaches, usually providing content curated by expert research teams, allow for immediate use and scalability but may fall short of generating sufficient interest and maintaining long-term use and be more suitable for social interaction [54] and activity-based applications such as iCST [33], music [55], and games [56]. The data obtained by digital technologies open up new opportunities to use machine learning to develop automation that can overcome these personalization barriers as well as optimize and adapt interventions. However, the lack of available high-quality and personalized data sources for people with dementia has limited progress in AI development for dementia care [35], especially for applications to support communication and engagement. To address this, Aikomi’s modular architecture facilitates the seamless capture of user data across key function domains: personal profile, content tagging, sequence ordering, and response analysis. This approach can combine the precision of the bespoke approach with the convenience of using the preprepared on-demand content. In this pilot study, data from the interviews and the provided content were used with the preprepared generic content to create bespoke programs in approximately 2 weeks, a reduction from the 6 to 8 weeks reported for digital storytelling [28]. In addition, it was possible to evaluate the performance of personalization using high-context data such as personal profiles, content and sequence attributes, and behavioral responses captured by the Aikomi platform. A related approach was reported by another personalized digital application called Scrapbook, which demonstrates the importance of obtaining multiple personal context–related data inputs to enable analysis [57]. Although AI system development was not the focus of this pilot study and personalization was conducted manually by the research team, this study demonstrates the potential of using the Aikomi platform as a tool to generate personalized data for AI development, and a preliminary investigation was conducted to create machine learning models to automate the personalization process, which is reported elsewhere [58]. In addition, chatbot technology, pioneered by a reminiscence intervention called ReminX [59], demonstrates an alternative trajectory for AI-driven personalization in dementia care.

Although no effects on BPSD were expected from this single-intervention study, the transient behavior changes reported by care staff for some persons (persons 1, 10, and 13) after using the Aikomi application suggest that there may be potential for investigating Aikomi to affect more lasting behavior changes related to BPSD. Although there is currently only a weak clinical evidence base to support the use of cognitive stimulation to manage BPSD, the use of personalized digital interventions embedded with data capture functions may offer the potential to not only create more effective therapeutics but also generate personalized monitoring data that can provide more robust clinical evidence. In the last few years, digital therapeutics (DTX) has emerged as a new category of regulatory-approved medical products that are distinct from drugs and medical devices [60,61]. To date, no DTX interventions for dementia have received regulatory approval, but several applications have been developed for supportive care [62]. DTX for dementia was pioneered by ReminX [59], which was designated as a breakthrough medical device by the Food and Drug Administration (FDA), and more recently by CST Assistant [63], which is a clinical evidence–supported CST-based game application that is now commercially available in Europe. Given the continuing challenges in developing effective and affordable drug therapies for dementia and BPSD, digital interventions for personalized cognitive stimulation such as Aikomi may have potential for clinical development as DTX and offer nondrug options for the management of BPSD and improvement in QOL. In addition to the primary objective of addressing therapeutic goals, the importance of preserving care relationships and creating opportunities for positive aspects of care for caregivers is also gaining increasing attention in dementia care [64,65]. With the growing shortage of professional caregivers, which is particularly acute in Japan, digital technologies that can promote meaningful engagement and improve QOL for both people with dementia and caregivers may become important tools to foster greater participation by family and informal caregivers in caring for their loved ones.

### Limitations

As this study was the first evaluation of the Aikomi application conducted in a care setting and limited to a single intervention to confirm its safety and acceptability for people with dementia, all inferences are preliminary and need to be confirmed via further multiple-intervention studies. Furthermore, although the aim was for Aikomi to be used independently by the care staff, the research team was required to be present during the interventions for this pilot study, which was a potential source of bias. This was necessary as the Aikomi platform was still a prototype, and the care staff were not familiar with operating digital technologies and required support to use Aikomi and overcome any technical difficulties. In fact, no application-related technical problems were encountered during the intervention, and connectivity issues were largely avoided by using tablets with SIM cards and addressed before the intervention. However, the provision of appropriate training and technical support for care staff to set up, use, and maintain the Aikomi application was not investigated in this pilot study. Another limitation was that most staff members were unfamiliar with conducting EPWDS and MENFIS evaluations, and the evaluations of some participants were conducted by multiple care staff members because of shift changes, which may have led to some inconsistencies in the results. Furthermore, the EPWDS is not yet available in Japanese, and an unvalidated translation prepared by the research team was used. These limitations need to be addressed in future studies.

### Future Research

The next step is to conduct multiple-intervention studies to investigate the effects of longer-term use of the Aikomi application when the program content of each intervention is adapted based on the behavioral responses of the person with dementia. The data derived from these studies will become the basis for developing machine learning models to create algorithms that can optimize personalization and increase the convenience for the care staff to use the system. To generate evidence to support the use of the Aikomi application in dementia care, it is necessary to conduct clinical trials to evaluate its effects on QOL and BPSD, from which its potential for further development as a digital therapeutic can be assessed. Separately, use by family caregivers in their own homes will be investigated to obtain feedback and data that will guide the development of the Aikomi application for community use. In addition, further work is needed to increase the pool of diverse and culturally relevant content that has been curated for people with dementia to reduce the lead time required to prepare stimulation programs. In this pilot study, all non–family-derived content was selected or created by the research team from publicly available sources, and copyright issues were not considered because of the noncommercial nature of this research. However, ensuring copyright compliance for the use of all digital media content, for both content owners and content providers, is a significant issue that must be addressed before the commercial deployment of personalized cognitive stimulation approaches. Finally, to enable more convenient use of smell stimuli with cognitive stimulation, automated smell delivery devices such as diffusers should be investigated for integration with the Aikomi platform.

### Conclusions

This pilot study demonstrated that the Aikomi application was able to create personalized cognitive stimulation programs that were acceptable for use in Japanese care homes and may have the potential to promote communication between people with dementia and their care staff. The use of smell stimuli paired with audiovisual stimulation was found to promote enjoyable interactions for many users. In addition, the Aikomi platform captured several types of personalized data, including the behavioral responses during the intervention, which enabled a detailed analysis of the stimulation content preferences of each person with dementia. These results indicate that the Aikomi application has the potential to be used as a tool to provide personalized cognitive stimulation and also generate high-context data suitable for the future development of AI systems to automate the personalization process. Further research will be conducted to develop the Aikomi application as a communication tool that can be easily used by nonspecialist care staff and families in residential and community care settings to enhance care relationships and positive aspects of care and aid the therapeutic management of BPSD.

## Appendix

Multimedia Appendix 1 Full details of engagement during the session with the Aikomi device.

## Abbreviations

AI: artificial intelligence
BPSD: behavioral and psychological symptoms of dementia
CMS: content management system
CST: cognitive stimulation therapy
DTX: digital therapeutics
EPWDS: Engagement of a Person with Dementia Scale
FDA: Food and Drug Administration
HDS-R: revised Hasegawa Dementia Scale
iCST: individual cognitive stimulation therapy
MENFIS: Mental Function Impairment Scale
MMSE: Mini-Mental State Examination
NM: Nishimura Mental State Scale for the Elderly
NPI-NH: Neuropsychiatric Inventory–Nursing Home version
QOL: quality of life

## Data Availability

The data sets generated and analyzed in this study are not publicly available as the data contain information that could compromise the privacy of the research participants. The data are available from the corresponding author upon reasonable request.
