# Supplementary material for: Enabling Personalization for Digital Cognitive Stimulation to Support Communication With People With Dementia: Pilot Intervention Study as a Prelude to AI Development
Source: JMIR Form Res. 2024 Jan 16;8:e51732. doi: 10.2196/51732 (PMC10828943; doi:10.2196/51732)
Supplement: Multimedia Appendix 1 [file formative_v8i1e51732_app1.docx]

**Appendix 1.** Full details of engagement during the session with the Aikomi device.

| Person | EPWDS^a^ | | | Caregiver written comment provided after the session (translation from Japanese)^b^ |
| --- | --- | --- | --- | --- |
|  | Positive engagement | Negative  engagement | Total score |  |
| 1 | 23 | 23 | 46 | Became very spontaneous for family and warship (themes). |
| 2 | 12 | 21 | 33 | Sang to childhood song (called “Furusato”). Concentrated on volleyball and knitting. Decreased memory of everything, unable to recall family faces and names. Severe memory loss symptoms. |
| 3 | 17 | 20 | 37 | Program stopped when she touched the screen. It is difficult for her to without carer support. Showed a quick response to music, she has excellent memory for sounds and often sang the lyrics correctly, especially for the chorus. However, she shows the same reactions when a tablet is used to listen to the songs. I thought her restlessness decreased a little. |
| 4 | 18 | 23 | 41 | She was quite sleepy at the beginning and did not seem to be in a good mood, but she was able to concentrate in her own way thanks to the music. She was very focused on Harumi Miyako (popular Japanese singer). Usually, she can`t concentrate for 5 minutes, it is very unusual for her to maintain concentration for 30 minutes. |
| 5 | 25 | 21 | 46 | At the start, he seemed negative because he said he couldn`t see, but when the photos started, he moved closer and said his son's name, and we could understand that he could see the images adequately. For the movies, he told us the name of the director and gave us very detailed information. Even when he was talking about something else, his eyes were focused on the tablet when the picture came on, which is something we don't see usually see in the facility. Talking about boxing with someone would be stimulating for him, but since this is not possible in his current situation the tablet could be effective. |
| 6 | 16 | 18 | 34 | Despite being a shy person, he was able to sing in front of everyone. He can`t name things and is not able to recognize things on a daily basis, but he could recognize some things in the video, such as a bottle. |
| 7 | 20 | 22 | 42 | She moved her hands to Kurodabushi (local folk song), Hawaiian, and spring childhood song. She did hand gestures of Japanese dance and hula dance by herself. She did not say much because she is a naturally reserved person, but she showed concentration and seemed excited. |
| 8 | 15 | 24 | 39 | She talked continuously unrelated to the pictures, even while looking at the tablet, but showed good autonomy and facial expression toward the music and smells. |
| 9 | 23 | 25 | 48 | Although he had lived with his mother in the past, he did not remember her and had never mentioned her before, but he said she had taken care of him. There were occasions when he explained to us in great detail about his old hobbies. Although currently he is not re-starting these hobbies, I think he might if we were to talk about them with him and this can be the catalyst for our efforts. He was anxious because he was not able to detect the smells. |
| 10 | 22 | 23 | 45 | She spontaneously said she wanted to do painting and lace making. For the mountains, her facial expression showed excitement. She appeared to be feeling good, and usually she does not continue to laugh, which is I think is due to the continued stimulation. She responded "I don't know" in response to a person, which I think means she doesn`t know the name rather than she doesn’t understand because she usually says "I don't know" to anything I ask her, such as seasons. |
| 11 | 24 | 21 | 45 | She hummed and clapping to the music, but she regularly shows almost the same behaviour to music such as karaoke. Although she could not correctly recognize the names of the people in the photos (she didn`t recognize her son), she showed very nostalgic comments, behaviours and emotions. |
| 12 | 20 | 20 | 40 | Usually, she is not a person who can show good concentration but was able to recall and talk about her past memories. Before the session she was agitated about something, but during the session she became calm and could concentrate. |
| 13 | 7 | 25 | 32 | She caught pneumonia just before the session which was lowering her will. She looked at the pictures but showed little response. |
| 14 | 18 | 22 | 40 | She showed most interest in the old photos. At the end of the session, she smiled and said "thank you". |
| 15 | 22 | 25 | 47 | She sang along to the music and looked nostalgic when watching the old photos. She smiled and pointed at me. |

^a^EPWDS: Engagement of a Person with Dementia Scale.

^b^Translation by the research team.

^c^DLB: dementia with Lewy bodies. Although the person was formally diagnosed with Alzheimer disease, the care staff indicated that she had a mixed presentation with DLB.
